# Brain food: rethinking food-borne toxocariasis

**DOI:** 10.1017/S0031182021001591

**Published:** 2022-01

**Authors:** Sara R. Healy, Eric R. Morgan, Joaquin M. Prada, Martha Betson

**Affiliations:** 1 Faculty of Health and Medical Sciences, School of Veterinary Medicine, University of Surrey, Guilford, UK; 2 Faculty of Health, Medicine and Life Sciences, School of Biological Sciences, Queen's University Belfast, Belfast, UK

**Keywords:** Food safety, human toxocariasis, neglected tropical disease, public health, zoonotic helminth

## Abstract

Human toxocariasis is a neglected tropical disease, which is actually global in distribution and has a significant impact on global public health. The infection can lead to several serious conditions in humans, including allergic, ophthalmic and neurological disorders such as epilepsy. It is caused by the common roundworm species *Toxocara canis* and *Toxocara cati*, with humans becoming accidentally infected *via* the ingestion of eggs or larvae. *Toxocara* eggs are deposited on the ground when infected dogs, cats and foxes defecate, with the eggs contaminating crops, grazing pastures, and subsequently food animals. However, transmission of *Toxocara* to humans *via* food consumption has received relatively little attention in the literature. To establish the risks that contaminated food poses to the public, a renewed research focus is required. This review discusses what is currently known about food-borne *Toxocara* transmission, highlighting the gaps in our understanding that require further attention, and outlining some potential preventative strategies which could be employed to safeguard consumer health.

## Graphical abstract

**Figure:**
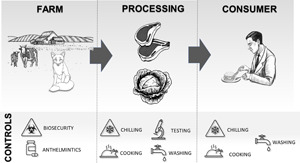

## Toxocariasis and food safety

Food safety is a major public health issue worldwide, and it is vitally important that any risks to consumers are managed both to protect the population and maintain consumer confidence. One such risk is the food-borne transmission of parasites to humans, which can have severe health implications for the global population. The most recently published assessment of the burden of food-borne parasites to humans estimated that 6.64 million disability-adjusted life years were lost due to the consumption of contaminated food in 2010 (Torgerson *et al*., 2015). Human toxocariasis affects an estimated 1.4 billion people worldwide (Ma *et al*., 2020). The disease is caused by *Toxocara canis* and *T. cati*, common roundworm parasites of canines and felines, respectively. *Toxocara vitulorum* affects bovine species and is generally believed to have less zoonotic significance, although knowledge of its transmission biology is currently lacking (Dewair and Bessat, 2020).

Human toxocariasis is recognized as one of the most commonly reported zoonotic helminth diseases worldwide (Magnaval *et al*., 2001; Nicoletti, 2013), having a significant impact on global public health (Zibaei and Sadjjadi, 2017). Toxocariasis can lead to a number of different clinical manifestations in humans, including allergic, ophthalmic and neurological disorders (Ma *et al*., 2018). Recent epidemiological studies and meta-analyses on cognitive impairment, psychosis and epilepsy have associated *Toxocara* infection with these debilitating neurological diseases (Walsh and Haseeb, 2012; Luna *et al*., 2018; Taghipour *et al*., 2021), and a potential link to the development of degenerative conditions such as Alzheimer's disease has been hypothesized (Fan, 2020). Despite recognition of its clinical impact, toxocariasis remains a neglected disease and major gaps in our understanding of the epidemiology of this parasite remain.

Large numbers of *Toxocara* eggs are excreted in the feces of infected dogs, cats and foxes (Morgan *et al*., 2013). Once present in the environment, these eggs can develop to the infective stage and persist in the soil for long periods (Fan *et al*., 2013). If infective eggs are consumed by accidental or paratenic host species, including humans, the larvae that hatch out in the gut subsequently migrate to several different organs in the body, becoming encapsulated in the tissues where larval development ceases. Studies have suggested it is primarily the host's immune response to *Toxocara* which is responsible for much of the pathology seen in cases of toxocariasis, rather than mechanical damage caused by migrating larvae (Epe *et al*., 1994). In cases of ocular infection, the response to a single larva can lead to vision loss (Neafie and Connor, 1976).

Whilst it is widely accepted that contaminated soil acts as a source of *Toxocara* eggs for human infections with potentially severe clinical consequences, little is known about food-borne transmission of this parasite. With eggs contaminating grazing pastures and growing crops, the pathway to food contamination appears wide open. However, this transmission route has received relatively little attention in the literature. *Toxocara* larvae are able to persist in the tissues of paratenic hosts, thus infected meat can act as a source of infection to humans (Strube *et al*., 2013; Wu and Bowman, 2020), particularly if consumed undercooked (Fan *et al*., 2015). A recently published review by Bowman (2021) discusses vegetable contamination with both *Ascaris* spp. and *Toxocara* spp. eggs (Bowman, 2021), but given that *Toxocara*, unlike *Ascaris*, cannot be transmitted by humans and has the potential to contaminate meat as well as vegetable produce, the epidemiology of this parasite warrants individual attention.

To establish the public health risks posed by *Toxocara* in food, a new research focus is now required. This review highlights the key knowledge gaps in our understanding of food-borne toxocariasis, assessing the evidence for this route of transmission by means of published human cases, serological studies and recovery of *Toxocara* spp. eggs and larvae from foods. The ability of current food safety measures to prevent toxocariasis is addressed, alongside discussion into future research avenues to determine appropriate prevention strategies for this overlooked, but potentially significant issue.

## Evidence of food-borne toxocariasis

### Human case studies

The reported cases of human toxocariasis attributed to food consumption identified in the literature are shown in Table 1. In all cases, the food product was reported to have been consumed raw or only partially cooked. Some had missing information about the patients’ age or the species of animal or type of tissue consumed. In all studies listed, the diagnosis of human toxocariasis was made by patient serology.

**Table 1.** Reported food-borne human toxocariasis cases in the literature

Country	Food type	No. of patients	Patient age/s (years)	Clinical presentation	Year	Reference
	*Meat products*					
Turkey	Unspecified raw meat	1	36	Unilateral neuroretinitis	2016	Karaca *et al*. (2018)
Japan	Chicken meat and bovine liver	1	60	Myelitis	2015	Hiramatsu *et al*. (2017)
Korea	Meat, liver, blood	5	30–60	Optic neuropathy	2014	Yang *et al*. (2014)
Korea	Bovine liver	1	46	Optic disc cyst	2013	Kim *et al*. (2013)
Korea	Cow meat, omasum, liver	1	51	OLM and VLM	2012	Park *et al*. (2012)
Korea	Bovine liver	1	35	Lung and liver abscesses, urticaria	2010	Kim *et al*. (2010)
Chile	Goat meat	1	51	Neurotoxocariasis and hepatopathy	2010	Finsterer *et al*. (2010)
Korea	Ostrich liver	1	17	Meningitis, lung and liver pathology	2010	Noh *et al*. (2012)
Japan	Deer meat	1	19	Myocarditis	2009	Enko *et al*. (2009)
Japan	Bovine liver	1	30	Pulmonary nodules	2008	Hisamatsu *et al*. (2008)
Japan	Bovine liver	3	58, 57, 27	Nodules in liver/lungs	2007	Yoshikawa *et al*. (2008)
Germany	Duck liver	1	55	Cerebral toxocariasis	2006	Hoffmeister *et al*. (2007)
Japan	Chicken liver	2	45, 71	Eosinophilia and pulmonary infiltrates	2006	Morimatsu *et al*. (2006)
Japan	Meat (species unknown)	1	26	Myocarditis	2002	Abe *et al*. (2002)
Korea	Dog liver and kidney	1	43	Pulmonary disease and eosinophilia	2002	Kim *et al*. (2002)
Japan	Bovine liver	1	26	Lung, liver and skin nodules	1999	Aragane *et al*. (1999)
USA	Ovine liver	1	63	Abdominal pain, cough, facial weakness	1992	Salem and Schantz (1992)
Japan	Equine, porcine, avian ‘meat’ + livers	2	52, 63	Liver granuloma	1990	Ishibashi *et al*. (1992)
Japan	Chicken liver and gizzard	2	22	Urticaria and hepatic enzyme elevations	1988	Nagakura *et al*. (1989)
Japan	Chicken and cow liver	3	39, 57, 46	VLM	1986	Ito (1986)
	*Blood products*					
Korea	Roe deer blood	1	58	Liver mass, cough, skin rash	2018	Park *et al*. (2018)
	*Gastropods and earthworms*					
Switzerland	Slugs	1	71	Neurological, ocular, pulmonary disease	2014	Fellrath and Magnaval (2014)
Italy	Snails	1	54	Encephalitis, neurological disease	2012	Caldera *et al*. (2013)
USA	Earthworm	1	16	Pulmonary nodules	2004	Cianferoni *et al*. (2006)
Spain	Snails	1	34	VLM, pulmonary disease	1991	Romeu *et al*. (1991)
	*Vegetables*					
Turkey	Salad	1	29	Peritonitis	2019	Arslan *et al*. (2019)
Romania	Raw vegetables	1	17	Eosinophilic ascites	2005	Chira *et al*. (2005)

Abbreviations: OLM = Ocular larva migrans, VLM = Visceral larva migrans.

When reviewing published toxocariasis case reports, one must remain open to the possibility that we are only seeing the tip of the iceberg. Toxocariasis can have non-specific symptoms, which may be attributed to other aetiologies and not investigated, leading to under diagnosis (Magnaval *et al*., 2001; Carlin and Tyungu, 2020). In many of the published case reports, details of the patients’ history were lacking: either no exposure history was taken by the clinician, the patient was not questioned on their dietary habits, or enquiries were only made regarding contact with animals. Cases were more commonly reported in countries where consumption of raw meat products is more widespread, an association noted elsewhere in the literature (Morimatsu *et al*., 2006).

### Larval distribution and persistence in animal tissues

Previous experimental studies have investigated the larval migration patterns for *Toxocara* spp. in various paratenic host species. In mice and rats, both *T. canis* and *T. cati* are initially detected in the liver and lungs during the so-called ‘hepato-pulmonary’ phase and are later found to undergo a ‘myotropic-neurotropic phase’, migrating to the skeletal tissues and brain (Abo-Shehada *et al*., 1984). In mice, *Toxocara cati* is reported to be less likely than *T. canis* to infect brain tissue, and more likely to be found in the musculature (Havasiová-Reiterová *et al*., 1995). Once present in these tissues, larvae have been found to persist for up to one year in mice (Bardón *et al*., 1994).

In rabbits experimentally infected with *T. canis* and *T. cati*, larvae could be recovered from the liver, lungs, kidneys and brain, with those in the liver being detected alive over 7 months post-infection; the musculature was not examined in this study (Pankavich, 1966). In piglets fed embryonated *T. canis* eggs, larvae were recoverable from the lungs and the brain, as well as in tissues more commonly consumed by humans including the kidneys, liver and musculature. Larvae collected from the livers 30 days post-infection and subsequently fed to recipient mice were able to induce infection, confirming larval viability (Sasmal *et al*., 2008). However, in a separate study, significant decreases in larval numbers were observed up to 3 weeks post-infection, suggesting that larvae seem to be less able to persist for long periods in the tissues of pigs (Helwigh *et al*., 1999). In lambs fed infective *T. canis* eggs, larvae were detected in the liver and lungs but muscles were not examined (Aldawek *et al*., 2002). An earlier study detected larvae in the muscles of lambs in addition to the pancreas, heart, kidney and brain, while larvae appeared to remain in the liver in year-old animals, not migrating to other tissues but remaining alive for the duration of the 12-week study (Schaeffler, 1960). *Toxocara* larvae within chicken tissues have been found to migrate to the lungs, liver, brain and muscles, with an ability to remain infective for prolonged periods of time. *Toxocara canis* larvae were found predominantly in chicken liver for up to 3.5 years (Tsvetaeva *et al*., 1979), whereas *T. cati* larvae appear to favour migration through the liver and lungs to the musculature, remaining infective to mice 176 days post-infection (Taira *et al*., 2011). Published studies in cattle are scarce, but a study reported the usual larval migration sites: liver, lungs, kidney and brain, in calves experimentally infected with *T. canis*. Muscle tissue was not examined for larvae in this study (Fitzgerald and Mansfield, 1970).

Given that larval migration to the liver is a common finding in the published literature, it is easy to see why so many of the human cases reported have been attributed to the consumption of this particular tissue. Moreover, experimental studies provide evidence of larval migration to other tissues consumed by humans, including the musculature. Thus, the consumption of raw or undercooked meat products, in particular liver tissue, is best avoided to reduce the risk of developing toxocariasis.

### Natural infections in food-producing animals

In addition to experimental studies, there are a limited number of reports where larvae have been detected in naturally infected animals. For example, *T. cati* has been detected in pig tissues intended for human consumption (Davidson *et al*., 2012) and both *T. canis* and *T. cati* have been isolated from naturally infected chickens (Zibaei *et al*., 2017; Okada *et al*., 2021). Indirect evidence of *Toxocara* infection in the form of serological data has also been published, with anti-*Toxocara* antibodies detected in several food-producing animals on farm and at slaughter. Lloyd (2006) found that up to 47% of sheep in a Welsh study had anti-*Toxocara* antibodies present in their blood, with levels directly proportional to animals’ age. This finding was supported by a 2011 Brazilian study, with 52.9% of female sheep between 11 and 15 months reported to have anti-*Toxocara* antibodies, compared with 5% sero-prevalence in lambs aged 0–6 months (Santarém *et al*., 2011). Serological investigations have also been undertaken for chickens, with 58.5% (Campos-da-Silva *et al*., 2015) and 67.7% (Oliveira *et al*., 2018) of birds in Brazil confirmed to have anti-*Toxocara* antibodies. Whilst circulating antibodies do not prove the presence of infective larvae in the tissues, they confirm exposure of animals to *Toxocara* spp. and indicate the presence of this parasite in farm environments and common exposure of food-producing animals, presumably by ingestion of infective eggs.

### Contamination of *Toxocara* eggs on farms

Several published studies have investigated the contamination of agricultural environments with *Toxocara* eggs. In Poland, 34.6% of soil samples from conventional farms and 21.3% from organic farms were positive for *Toxocara* spp. eggs (Klapec and Borecka, 2012). In the Philippines, the overall prevalence of *Toxocara* in soil samples was much lower at 4%, with no statistically significant difference reported between conventional and organic farms (Paller and Babia-Abion, 2019). Eggs may be transferred in contaminated soil to vegetable crops destined for human consumption. In their study, Klapec and Borecka (2012) analysed various vegetable produce harvested with the surrounding soil, detecting eggs on 10.8% of the sampled produce from organic farms, and 19.2% from conventional farms (Klapec and Borecka, 2012). An Iranian study found that overall, 3.97% of sampled vegetables were contaminated with *Toxocara* eggs (Fallah *et al*., 2016), slightly higher than the 1.68% prevalence reported in another study from the same country (Rostami *et al*., 2016) and the 1.5% prevalence reported in Turkey (Kozan *et al*., 2005). It is difficult to compare the results of these studies due to variations in sampling strategies and laboratory techniques, but these studies confirm the link between *Toxocara* eggs in the soil and contamination of vegetable produce.

*Toxocara* eggs can also reach people from soil or feces *via* invertebrates. There are reported human cases arising from gastropod ingestion, and one case from earthworm ingestion (Table 1) (Romeu *et al*., 1991; Cianferoni *et al*., 2006; Fellrath and Magnaval, 2014). Whilst this situation is probably extremely rare, one must bear in mind that contamination of vegetables with common invertebrates such as these does occur and could pose a risk to consumers if produce is consumed unwashed. Cases of zoonotic disease from accidental ingestion of molluscs infected with nematodes such as *Angiostrongylus cantonensis* (Slom *et al*., 2002) attest to this possibility, while the possible growth of insects as human food in future (Babarinde *et al*., 2020; Hawkey *et al*., 2021) demands consideration of their potential as sources of zoonoses, including *Toxocara*. Insects are known to transfer taeniid cestode eggs from feces to food (Benelli *et al*., 2021), although this route has not yet been demonstrated for *Toxocara* spp.

## Sampling techniques and diagnostics for *Toxocara* spp.

### *Toxocara* eggs in soil and vegetable produce

Most of the published methods for the recovery of *Toxocara* sp. eggs from soil and vegetables are based on conventional techniques, involving steps such as washing, sieving, sedimentation, filtration and flotation prior to microscopic examination. In some cases, egg viability determination is also undertaken, usually by means of dye uptake differentiation (Dabrowska *et al*., 2014). Sample sizes vary widely between studies. In the case of soil, from 3 g up to 200 g of dried sample has been used, which is typically extracted up to 5 cm from the soil surface (Paller and Babia-Abion, 2019; Tyungu *et al*., 2020). For vegetable testing, preferred weights of samples usually range from 100 to 250 g, with the processing of sedimented washing solution and subsequent residue concentration commonly undertaken prior to parasitological analysis *via* microscopy (Abougrain *et al*., 2010; Fallah *et al*., 2016; Hajipour *et al*., 2021). Some studies have additionally utilized PCR-based molecular assays, to differentiate between *T. canis* and *T. cati* eggs in soil samples (Choobineh *et al*., 2019; Tyungu *et al*., 2020) and vegetable produce (Guggisberg *et al*., 2020).

### *Toxocara* spp. larvae in meat tissues and animal serology

In order to recover *Toxocara* larvae from meat products, the tissue is usually subjected to chemical digestion treatment using an HCl-pepsin solution with simultaneous incubation and mechanical stirring, followed by sedimentation, filtration and identification of larvae by microscopy. Subsequent molecular analysis of the larvae obtained using PCR-based techniques is commonly undertaken following isolation (Zibaei *et al*., 2017; Okada *et al*., 2021).

For the purpose of identifying animals with circulating anti-*Toxocara* antibodies in their bloodstream, studies have utilized enzyme-linked immunosorbent assay (ELISA) based detection using the excretory-secretory (TES) antigens of *T. canis* larvae. To reduce cross-reactivity with *Ascaris* spp. and improve test specificity, serum samples are typically pre-adsorbed with the extract of an adult *Ascaris* worm prior to ELISA testing (Santarém *et al*., 2011; Rassier *et al*., 2013; Campos-da-Silva *et al*., 2015). No attempt is usually made to differentiate between *T. canis* and *T. cati* infections in these assays, and cross-reactivity between these species is likely (Santarém *et al*., 2011).

Whilst the presence of circulating anti-*Toxocara* antibodies does not definitively diagnose an active *Toxocara* infection, it is suggestive of exposure to this parasite. In contrast, identification of *Toxocara* spp. eggs in the feces of infected cats, dogs and foxes is more suggestive of a patent infection, with adult worms residing within the intestine of these definitive hosts (Fan, 2020). The risk of a false-positive diagnosis due to the ingestion of faeces containing *Toxocara* spp. eggs can be reduced with the addition of ELISA-based coproantigen detection (Elsemore, 2020), or repeat sampling following the gut transit time.

### Diagnosing human toxocariasis

In the case of human *Toxocara* infections, diagnosis is most commonly based on the results of clinical and serological findings. As is the case for animal serological testing, ELISA-based tests are utilized to detect TES antibodies in the blood of human patients. As a confirmatory measure, following a positive ELISA result, Western blotting techniques are recommended which improve the sensitivity and specificity of diagnosis (Mazur-Melewska *et al*., 2020). Research to develop improved serodiagnostic assays for *Toxocara* sp. is ongoing. The use of recombinant antigens and the detection of antibody subclasses are approaches currently being explored for their potential to improve the reliability of testing for toxocariasis in humans in the future (Nicoletti, 2020). Improvements in the diagnosis of *Toxocara* spp. infections in humans would help to elucidate dietary risk factors by enabling larger and more precise epidemiological studies.

## Control measures to minimize transmission risk

**Fig. 1.**
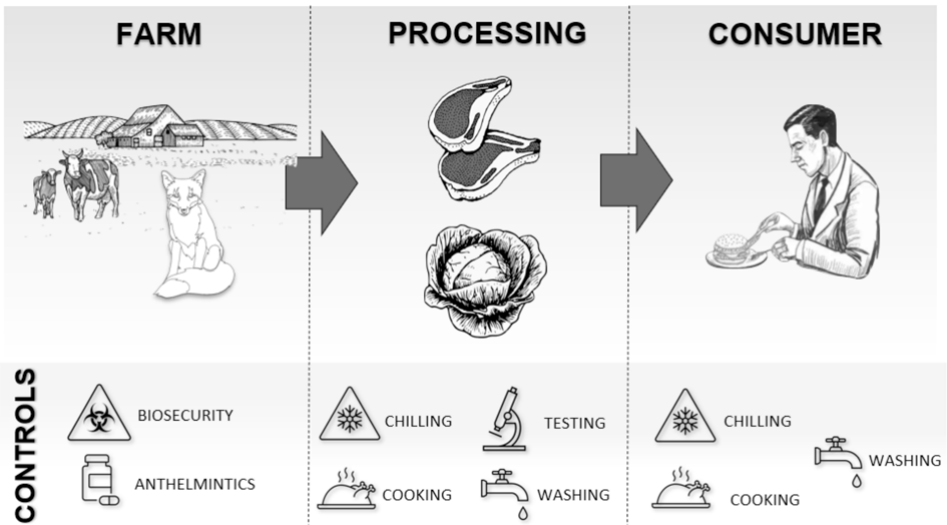
The potential flow of *Toxocara* spp. from the farm to the consumer, and a summary of the possible control measures at each step of the production chain.

The potential flow of *Toxocara* spp. from the farm to the consumer, and a summary of the possible control measures at each step of the production chain are summarized in Fig. 1. While many food systems have steps in place to protect consumers from other pathogens, their effectiveness against *Toxocara* spp. has not been systematically tested.

### Farm biosecurity

*Toxocara* spp. eggs are deposited in the soil *via* the feces of infected dogs, cats and foxes, and once present in the soil can remain infective for years due to their resistance to environmental conditions (Parsons, 1987). Because organic fertilizer does not typically incorporate the feces of these definitive hosts, its agricultural use should not usually pose a risk of contaminating the environment with the eggs of this parasite. No practical methods exist to remove *Toxocara* eggs from the environment (Overgaauw and van Knapen, 2013); therefore, controlling access of these definitive hosts to agricultural land is a logical first intervention point to explore for both meat and vegetable production processes.

An Italian study which assessed the level of *Toxocara* eggs in the soil of farms in the Marche region found that out of 60 farms sampled, around 50% had soil that tested positive for *Toxocara*. In addition, the proportion of positive farms was almost twice as high in the group which had more than three dogs living on-site, compared to farms that had three dogs or fewer (Habluetzel *et al*., 2003). Although scarce, other studies have also found *Toxocara* spp. on livestock farms. These include a 2002 study, which assessed a large-scale poultry unit and its surrounding environment in Poland, detecting *Toxocara* spp. eggs in soil within close proximity to the farm (Trawinska *et al*., 2002), and a study in 2003 which assessed the environmental microbial composition of two pig farms in Poland, detecting *Toxocara* spp. eggs in the soil of both units sampled (Szostak and Bekier-Jaworska, 2003).

There are other studies in the literature which have focused on determining the prevalence of *Toxocara* spp. in definitive hosts that could access agricultural land. For example, some authors have investigated *Toxocara* prevalence in free-roaming farm cats by faecal analysis: *T. cati* was detected in 91% of sampled cats in the UK (Yamaguchi *et al*., 1996), whereas an earlier study obtained positive results in 63% of cats (Gethings *et al*., 1987). In farm dogs, a Portuguese study sampling canine feces on 165 small-ruminant farms detected a *Toxocara* prevalence of 8% using microscopic evaluation (Cardoso *et al*., 2014), and studies assessing *T. canis* infection in foxes found prevalence levels of 47.4% in Zurich, Switzerland (Hofer *et al*., 2000); and 55.9% in the UK (Richards *et al*., 1995). It is always difficult to compare the results of studies such as this directly, because of variations in sampling techniques and laboratory methods. In these studies, the soil was not analysed, but infected animals potentially had access to farmland, in particular land utilized for arable production or livestock grazing. In the previously referenced study reporting a 47% sero-prevalence of *T. canis* in Welsh sheep, all participating farms had dogs resident on-site, and foxes were also present (Lloyd, 2006). In addition, stray cats of unknown health status were present on farms from which *T. cati* larvae were isolated from pig meat destined for human consumption in Norway (Davidson *et al*., 2012), and chicken meat from a commercial unit in Japan (Okada *et al*., 2021). As well as defecating onto agricultural land, there is the potential for definitive hosts, mainly cats, to contaminate animal feed stores. This route of infection has been seen in cases of toxoplasmosis in pigs, a parasitic infection which shares several features with toxocariasis including a common definitive host (Li *et al*., 2010).

Regular treatment of farm cats and dogs with appropriate anthelmintic drugs in accordance with guidelines such as those of the European Counsel for Companion Animal Parasites (ESCCAP) is one approach to reducing the contamination of the agricultural environment with *Toxocara* eggs, particularly for kittens, pups and nursing queens and bitches (Overgaauw and van Knapen, 2013). A 2016 study by Nijsse reported that fewer than a quarter of cat owners questioned were treating animals with an anthelmintic at the recommended frequency, with a higher risk of *Toxocara* shedding in free roaming cats (Nijsse *et al*., 2016). Treating owned animals is just one part of the picture. A meta-analysis showed the prevalence of *Toxocara* in stray cats, working and rural dogs to be significantly higher than that of pet cats and dogs (Rostami *et al*., 2020), and parasites in foxes should also be considered (Deplazes *et al*., 2004).

In a recent study, Hajipour *et al*. (2021) assessed the impact of fencing-off farmland on the contamination of vegetables with *T. canis* and *T. cati* in Iran and found unfenced cropping areas had a vegetable contamination rate of 55.3% compared to 9.2% in fenced areas (Hajipour *et al*., 2021). Whilst this control step seems logical, the size of the farms is important to consider: in this study, the largest site was 20 hectares, considerably smaller than the average farm size of 87 hectares in England, UK (Defra, 2021). Farm size could impact the feasibility and economic viability of biosecurity measures such as fencing.

### Meat inspection at slaughter

In terms of reducing the risk of parasitic transmission to humans, meat inspection is currently the principal means of controlling *Trichinella* spp. and *Taenia* spp. infections in the food chain (Dorny *et al*., 2009). At this time, the presence of *Toxocara* spp. larvae in the tissues of food animals is not assessed at slaughter in any jurisdiction. Indeed, *Toxocara* larvae are frequently seen as a ‘contaminant’ when isolated during routine porcine *Trichinella* testing by artificial tissue digestion (Marucci *et al*., 2013), and their presence is often not reported. Some recent studies have advocated the use of molecular methods to improve the detection of helminth contamination of meat and offal (Nguyen *et al*., 2017; Wang *et al*., 2018; Karadjian *et al*., 2020). This is an area which has the potential to greatly improve the detection of *Toxocara* during the meat inspection process and warrants further investigation.

### Washing vegetable produce

The contamination of vegetable produce at source and human consumption without washing is a potential transmission route for several parasites, including *Toxocara* spp. (Slifko *et al*., 2000; Mosayebi *et al*., 2014). Several published studies have assessed the degree of contamination of vegetable produce with the eggs of *Toxocara* spp. worldwide, with leafy vegetables such as salad leaves seemingly more susceptible compared to other vegetable types. For example, in Iran, Hajipour *et al*. (2021) reported 40.2 and 33.0% of lettuce samples tested to contain the eggs of *T. cati* and *T. canis* respectively, compared with radishes from which only *T. cati* was isolated in 6.1% of samples (Hajipour *et al*., 2021). In Libya, *T. canis* and *T. cati* eggs were detected in 37 and 48% of lettuces compared to 14 and 8% of cucumbers, respectively (Abougrain *et al*., 2010). The higher levels of contamination reported on leafy vegetables could be due to an increased surface area in contact with the contaminated soil surface (Gupta *et al*., 2009; Maikai *et al*., 2012). Studies have assessed the effectiveness of washing different vegetables in reducing parasitic contamination. Avcioglu *et al*. (2011) did not detect any helminth eggs in vegetable samples following a washing step with clean water alone (Avcioglu *et al*., 2011), a finding supported by other studies in the literature (Fallah *et al*., 2012; Rostami *et al*., 2016). Alternative washing methods for the removal of parasitic species from vegetables have been investigated, with 0.95% calcium hypochlorite solution found to be more effective at reducing parasitic contamination than 1% lemon juice, 1% vinegar or a diluted dishwashing detergent (Hajipour *et al*., 2021). However, consumer safety and public acceptance of chemical food treatments need to be considered. In some countries, for example, the use of chlorine-based disinfectants for food produce is highly restricted or not authorized (De Corato, 2020).

### Chilling and freezing of foods

Refrigeration is used throughout the food chain, from the production stage through to the consumer, to maximize food quality and safety (Tassou *et al*., 2010). However, there are very few published studies assessing the effect of chilling and freezing on the viability of *Toxocara* spp. eggs and larvae, particularly in food products. A study by Taira *et al*. (2011) demonstrated that the infectivity of *T. cati* larvae within chicken muscle tissue reduced substantially following chilling at 4°C for 14 and 28 days, but larvae were still viable and induced infections in recipient mice (Taira *et al*., 2011). In a similar study, chilling pig and poultry tissues at 4°C for 7 days had a significant effect on larval infectivity, but again infections could still be established in recipient pigs (Taira *et al*., 2004). Chilling infected mouse liver samples to between 0 and 4°C for 10 days was found to significantly reduce the intensity of infection in recipient mice (Dutra *et al*., 2013). These studies suggest the potential risk of *Toxocara* transmission by consumption of meat products is decreased by refrigeration, but the risk of human infection remains.

Freezing of meat tissues appears to have a more significant effect on the viability of *Toxocara* larvae compared to refrigeration. Storing infected muscle tissue at −21°C for 12–48 h was found to have a dramatic impact on larval viability, with no subsequent infections detected in recipient mice. Larval motility was reported as absent after 24 h freezing at −25°C, suggesting that the thermal death point for the larvae had been reached (Taira *et al*., 2011). This finding was supported by Dutra *et al*. (2013), with a 100% reduction in larval viability following freezing of mouse liver tissue at −20°C, with larvae showing detrimental morphological changes including ruptured cuticles and internal organ degeneration (Dutra *et al*., 2013). In contrast, Sprent (1953) reported finding motile larvae in mice carcasses subjected to −20°C for 4 weeks, although the viability of these larvae was not assessed (Sprent, 1953). Freezing meat products is a promising potential control point to investigate, and is utilized for some other food-borne parasites such as *Trichinella spiralis* (Noeckler *et al*., 2019). This would be especially important for meat intended to be served in raw dishes, such as steak tartare. However, effects on food quality and palatability brought about by the freezing process need to be considered if this control measure is implemented during processing (Zhang *et al*., 2019).

The environmental resistance of *Toxocara* spp. eggs is widely reported (Mizgajska, 2001; Despommier, 2003), with studies demonstrating the tolerance and development of eggs at a range of ambient temperatures (Azam *et al*., 2012). A study by Azam *et al*. (2012) assessed the impact of low temperatures on *Toxocara* eggs. *Toxocara canis* eggs were stored at +1 and −2°C for a 6-week period, during which time they did not embryonate to become infective, and their subsequent development was delayed once returned to higher temperatures (Azam *et al*., 2012). Whilst consumers are unlikely to routinely store vegetables for this length of time in domestic refrigerators, storing vegetable produce at low temperatures could postpone the development of eggs such that any consumed are less likely to be fully larvated and thus infective. Freezing eggs was found to reduce the viability of *T. canis* and *T. cati*, with longer treatment times correlating with reduced viability (O'Lorcain, 1995). As is the case for meat products, the process of freezing vegetable produce can lead to structural changes and a reduction in product quality, with some types of vegetable more negatively impacted by freezing than others (Jeremiah, 2019).

### Cooking foods

Heat treatment remains one of the most reliable methods to inactivate food contaminated with parasites, such as *Taenia* spp., *Toxoplasma gondii* and some *Trichinella* spp., provided the internal temperature is high enough (Franssen *et al*., 2019). For example, in the case of *Taenia solium* infected pork, cooking for 10 min at a core temperature of 80°C, 20 min at 70°C or 30 min at 60°C has been found to kill cysticerci, but at core temperatures of 40°C, metacestodes were still viable after up to 1 h of cooking. This raises some concerns for faster cooking methods, such as deep frying (Møller *et al*., 2020). In comparison, *T. gondii* and *T. spiralis* appear to be more sensitive to heat treatment, with *T. gondii* tissue cysts killed following exposure to temperatures above 56°C for 10 min (Dubey *et al*., 1990), and a 15 min cooking time at 55.6°C sufficient to inactivate *T. spiralis* larvae in pork (Noeckler *et al*., 2019).

Publications outlining the effect of the cooking process on *Toxocara* spp. larvae in animal tissues and eggs on vegetable produce are currently lacking. A 2007 study assessed the infectivity of *T. canis* larvae present in mouse liver following cooking for 5 min in a household microwave, with the internal temperature of the tissue reaching >70°C. Transmission of *Toxocara* to recipient mice only occurred if infected liver was consumed raw (Cetinkaya *et al*., 2007). To assess this control step further, it would be necessary to determine precise thermal death curves (time *vs* temperature) for *Toxocara* spp. tissue larvae. This would be especially useful for meat more commonly served ‘rare’, such as beef, and to assess the risk of cooking methods more commonly associated with under-cooking, such as barbequing.

## Conclusions and future directions

Human toxocariasis is a neglected disease which has a significant negative impact on global public health. It has been known for some time that people can acquire *Toxocara* by ingesting larvated eggs from the environment, but there is also mounting evidence for food-borne transmission of this parasite. Seropositivity and viable larvae in food animals and *Toxocara* eggs on vegetable produce have been demonstrated and are of great concern, especially as current food safety measures do not specifically take this parasite into consideration. However, current knowledge of the true importance of food consumption in the epidemiology of toxocariasis is lacking. Further research is now required to quantify the flow of *Toxocara* from the environment to the final food product and evaluate the potential impact of any control interventions. A multifaceted approach will be required to address the different steps in the food production pathway, with molecular techniques opening the doors to more sensitive detection methods for *Toxocara* species to support both research and food safety. A renewed research focus is urgently required to fill in the key gaps, inform food safety policy and, ultimately, protect consumer health.
